# Synthesis and Characterization of Acrylamide/Acrylic Acid Co-Polymers and Glutaraldehyde Crosslinked pH-Sensitive Hydrogels

**DOI:** 10.3390/gels8010047

**Published:** 2022-01-09

**Authors:** Munir Ahmad Khan, Abul Kalam Azad, Muhammad Safdar, Asif Nawaz, Muhammad Akhlaq, Pijush Paul, Md. Kamal Hossain, Md. Habibur Rahman, Roua S. Baty, Attalla F. El-kott, Mohamed Kamel, Simona G. Bungau, Mohamed M. Abdel-Daim

**Affiliations:** 1 Department of Pharmaceutics, Faculty of Pharmacy, Gomal University, Dera Ismail Khan 29050, Pakistan; muneermarwat305@gmail.com (M.A.K.); safdarlaghari10@gmail.com (M.S.); asifnawaz676@gmail.com (A.N.); dr.akhlaq@gu.edu.pk (M.A.); 2 Pharmaceutical Technology Unit, Faculty of Pharmacy, AIMST University, Bedong 08100, Kedah, Malaysia; 3 Department of Pharmacy, Gono Bishwabidyalay, Mirzanagar, Savar, Dhaka 1344, Bangladesh; pijushpaul8@gmail.com; 4 Institute of Health and Sports, Victoria University, Melbourne 3011, Australia; hossain_238@yahoo.com; 5 Department of Global Medical Science, Wonju College of Medicine, Yonsei University, Wonju 26426, Gangwon-do, Korea; pharmacisthabib@gmail.com; 6 Department of Biotechnology, College of Science, Taif University, P.O. Box 11099, Taif 21944, Saudi Arabia; rsbaty@tu.edu.sa; 7 Biology Department, Faculty of Science, King Khalid University, P.O. Box 9004, Abha 61421, Saudi Arabia; elkottaf@kku.edu.sa; 8 Zoology Department, Faculty of Science, Damanhour Univesity, Damanhour 22511, Egypt; 9 Department of Medicine and Infectious Diseases, Faculty of Veterinary Medicine, Cairo University, Giza 12211, Egypt; m_salah@cu.edu.eg; 10 Department of Pharmacy, Faculty of Medicine and Pharmacy, University of Oradea, 410087 Oradea, Romania; simonabungau@gmail.com; 11 Department of Pharmaceutical Sciences, Batterjee Medical College, P.O. Box 6231, Jeddah 21442, Saudi Arabia; 12 Pharmacology Department, Faculty of Veterinary Medicine, Suez Canal University, Ismailia 41522, Egypt

**Keywords:** hydrogels, pH-sensitive, colon drug delivery, acrylamide, acrylic acid, glutaraldehyde

## Abstract

This project aims to synthesize and characterize the pH-sensitive controlled release of 5-fluorouracil (5-FU) loaded hydrogels (5-FULH) by polymerization of acrylamide (AM) and acrylic acid (AA) in the presence of glutaraldehyde (GA) as a crosslinker with ammonium persulphate as an initiator. The formulation’s code is named according to acrylamide (A1, A2, A3), acrylic acid (B1, B2, B3) and glutaraldehyde (C1, C2, C3). The optimized formulations were exposed to various physicochemical tests, namely swelling, diffusion, porosity, sol gel analysis, and attenuated total reflection-Fourier transform infrared (ATR-FTIR). These 5-FULH were subjected to kinetic models for drug release data. The 5-FU were shown to be soluble in distilled water and phosphate buffer media at pH 7.4, and sparingly soluble in an acidic media at pH 1.2. The ATR-FTIR data confirmed that the 5-FU have no interaction with other ingredients. The lowest dynamic (0.98 ± 0.04% to 1.90 ± 0.03%; 1.65 ± 0.01% to 6.88 ± 0.03%) and equilibrium swelling (1.85 ± 0.01% to 6.68 ± 0.03%; 10.12 ± 0.02% to 27.89 ± 0.03%) of formulations was observed at pH 1.2, whereas the higher dynamic (4.33 ± 0.04% to 10.21 ± 0.01%) and equilibrium swelling (22.25 ± 0.03% to 55.48 ± 0.04%) was recorded at pH 7.4. These findings clearly indicated that the synthesized 5-FULH have potential swelling characteristics in pH 6.8 that will enhance the drug’s release in the same pH medium. The porosity values of formulated 5-FULH range from 34% to 62% with different weight ratios of AM, AA, and GA. The gel fractions data showed variations ranging from 74 ± 0.4% (A1) to 94 ± 0.2% (B3). However, formulation A1 reported the highest 24 ± 0.1% and B3 the lowest 09 ± 0.3% sol fractions rate among the formulations. Around 20% drug release from the 5-FULH was found at 1 h in an acidic media (pH1.2), whereas >65% of drug release (pH7.4) was observed at around 25 h. These findings concluded that GA crosslinked 5-FU loaded AM and AA based hydrogels would be a potential pH-sensitive oral controlled colon drug delivery carrier.

## 1. Introduction

The worldwide prevalence of colonic disease is increasing with over one hundred thousand colorectal cancer patients newly diagnosed every year [1]. Traditional non-targeted therapy has unwanted side-effects and offers low efficacy owing to the systemic absorption of the drug before it reaches the disease site [2]. Therefore, colon-targeted drug delivery systems for the local treatment of colonic diseases are urgently sought [3]. Depending on the colonic milieu, colon targeted drug delivery systems specifically release the drug where it is needed, thus circumventing the untimely release of the drug in the upper gastrointestinal (GI) tract [4]. It is important to take into consideration the area neighboring the disease site(s) as well as the colon physiology, since the GI tract experiences constant changes in motility, fluid composition, pH from the small to the large intestine, and enzymatic activity [5]. Numerous formulation strategies have been investigated to develop colonic drug delivery (i.e., enzyme-sensitive systems, magnetically triggered systems, and pH-responsive systems) [6]. Hydrogels are a three-dimensional network of crosslinked polymer that can absorb and hold a significant amount of water inside the gap between the chains [7]. Hydrogels are extensively explored in a wide range of applications including medical, biological, and pharmaceutical fields due to their high-water content, swelling characteristics, biocompatibility, high permeability, and nontoxicity [8]. Besides this, stimuli-sensitive hydrogels are gaining attention as smart drug delivery systems as they can respond to the surrounding environment (e.g., temperature, pH, and enzymes). pH-sensitive hydrogels are preferable to other systems due to their higher controllability [9,10]. Several types of pH-responsive hydrogels have been investigated for site specific drug delivery owing to significant pH variations in the GI tract, where the drug release characteristics change with the variations in the medium pH because of relaxation of the chain [11].

AA is a pH-sensitive polyelectrolyte, which is superabsorbent as well as possessing concentration-dependent mucoadhesive properties and has been exclusively explored in colon drug delivery systems [12]. The advantage of AA is that variation in the composition of the polymers results in different drug release characteristics depending on the surrounding pH [13]. Temperature-sensitive and pH-responsive behavior has also been displayed by the copolymers AA and the interpenetrating networks (IPNs) [14]. Also, pH-sensitive macromolecular structures designed by the poly(AA) (PAA) have been explored for use in site-specific drug delivery systems [15]. Owing to the ionization of the carboxylic acid (CA) functionalities, the PAA based hydrogels swell considerably at pH 7.4 since its pKa value ranges from 4.5–5 [16].

AM has been employed in various biomedical applications including protein drug delivery due to its biocompatibility, pH-responsiveness and mucoadhesivity [17]. Success has been reported using an AM grafted N-succinyl chitosan-based hydrogel with a pH dependent release of insulin when administered orally [18].

GA is used as a crosslinking agent for the preparation of hydrogels [19]. It is also employed for cell and enzyme immobilization due to its higher aqueous solubility, ease of accessibility, low cost and efficiency of biomaterials stabilization [20]. GA contains two aldehyde groups which can form immediate covalent bonds with the functionalities available (i.e., imidazoles, phenols, hydroxyl, and thiols) [21]. It has been reported that at a pH of around 7, GA reacts more quickly with amine groups [22] as well as forming better chemical and thermal crosslinks than those of other aldehydes [23].

5-FU is frequently used as one of the mainstays in potential antitumor drugs in clinical chemotherapy, specifically for the treatment of colon cancer [24]. It has a short biological half-life (10–20 min), causing partial absorption where? Upper intestine? since it is a sparingly water-soluble pyrimidine antimetabolite. The drug is rapidly metabolized by dihydro pyrimidine dehydrogenase after oral delivery [25]. The clinical use of 5-FU affects the normal cells, leading to the adverse effects on GI tracts, the central nervous system and bone marrow, owing to the lack of tumor selectivity [26,27]. In recent studies, pH-sensitive hydrogels have been investigated by researchers for controlled delivery of 5-FU to reduce these side effects and to enhance the antitumor activity [28]. PAA and its derivatives-based pH-responsive hydrogels can circumvent the effect of the highly acidic surroundings on drugs [29]. The pKa of PAA is around 3.5, therefore at low pH, the hydrogels based on PAA become contracted resulting in the premature release of the drug in the stomach. Consequently, at higher pH, they remain swollen, leading to drug release in the intestine. Nevertheless, avoiding premature drug release before entering the colon section is very challenging due to the pH resemblance between the small intestine and the colon [30].

The goal of this study is to synthesize pH-responsive hydrogels using AM, AA and GA. Hydrogels were prepared by a simple crosslinking method, employing 5-FU as a model drug. The ATR-FTIR spectroscopy was used to investigate the interaction between the drug and the excipients. Swelling characteristics, diffusion coefficient, drug loading, in vitro release and release kinetics were also studied.

## 2. Results and Discussion

### 2.1. Synthesis Hydrogel

**Scheme 1:**
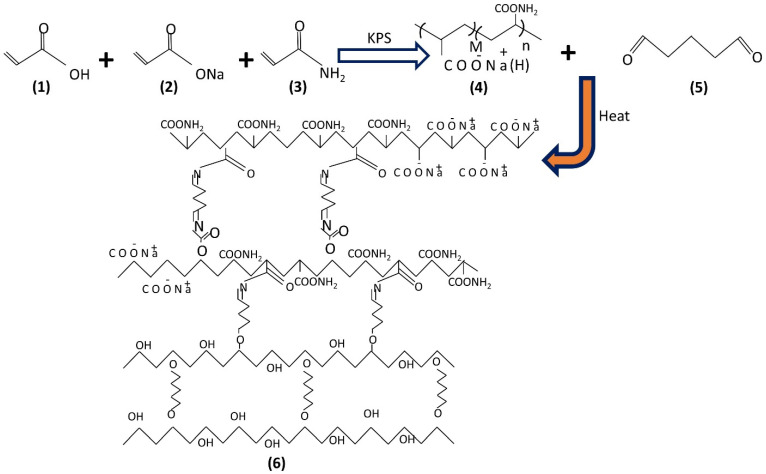
General mechanism for polymerization of AA and AM in the presence of glutaraldehyde cross-linker [31].

The mechanism of polymerization of acrylic acid and acrylamide in the presence of glutaraldehyde (GA) cross-linker was adapted from Wang et al. and partially modified the synthesis reaction mechanism shown in Scheme 1. Acrylic acid (1), acrylic acid sodium (2) and acrylamide (3) can react to Poly(acrylic acid-co-acrylamide) (4) using KPS as a radical initiator. The persulfate initiator was decomposed under heating to generate sulfate anion radical. The cross-linker glutaraldehyde (5) was added after polymerization finished in order to avoid the crosslinking reaction of Poly(acrylic acid-co-acrylamide) (4) during polymerization. Through heat-treatment at 110–160 °C the amide groups and hydroxyl groups can react with aldehyde groups from glutaraldehyde (5) to form poly(AA-co-AM) (6) network structure. Grafted hydrogels exhibit pH-sensitive swelling properties and pH-controlled drug release behavior.

### 2.2. Solubility Study

The 5-FU is a nucleobase analogue having a pKa value of (8 ± 0.1). It has a water solubility of 12.2 g/L at 20 °C. (repeated) It is freely soluble in a phosphate buffer of pH 7.4 as well as in distilled water compared to pH 1.2 (Table 1). The solubility study is an important parameter which conforms to the dissolution experiments.

### 2.3. FTIR

**Figure 1:**
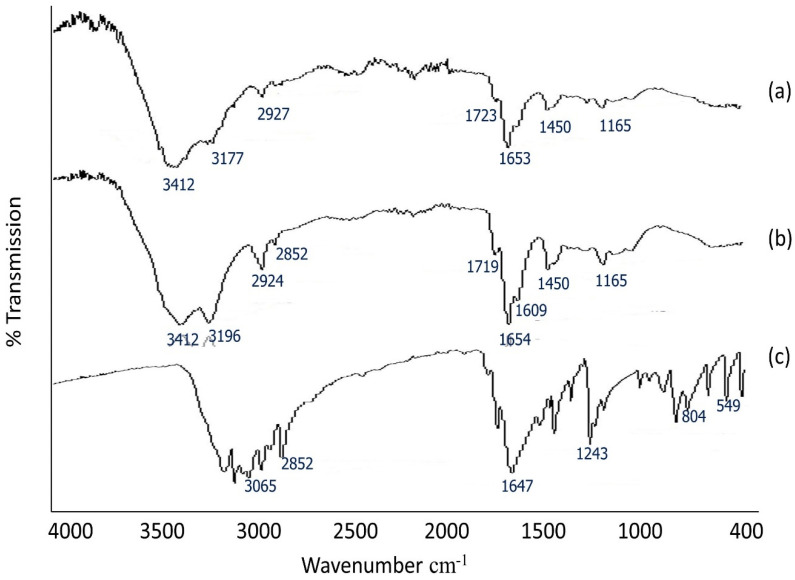
ATR-FTIR spectra of (**a**) Pure 5-FU, (**b**) 5-FULH, and (**c**) Unloaded hydrogels.

FTIR spectral analysis of 5-FU and 5-FULH is shown in Figure 1. Pure 5-FU, drug loaded, and unloaded hydrogel samples were assessed using FTIR in the array of 400–4000 cm^−1^. FTIR represents the distinct peak at 3177 cm^−1^, 2927 cm^−1^, 1658 cm^−1^ in pure 5-FU spectra and 3196 cm^−1^, 2924 cm^−1^ and 1654 cm^−1^ in 5-FULH. In the case of pure 5-FU, the bands at 3412 cm^−1^, 1165 cm^−1^ show evidence of N-H stretching (free) and –C-F band; after drug loading some of the bands disappear and N-H stretching (free) appears at the same wave number, while –C-F band appears at 1165 cm^−1^. This indicates that the 5-FU is molecularly dispersed into the prepared hydrogels [32]. In the case of unloaded hydrogel, see Figure 1c, the AM peak appears at 1647 cm^−1^ for primary amide (C=O) stretching. The peak for the alkene group (HC=CH) appears at 3065 cm^−1^. In the band of GA, the peak assigned to the C-OH stretching at 1243 cm^−1^ is very clear, suggesting that the desired material has been successfully prepared [33]. Owing to the electrostatic interaction among the various functional groups of AM, AA and GA, the characteristic peaks of AM, AA and GA are shifted to developed 5-FULH. Figure 1b shows that the loading of 5-FU by the developed hydrogel was successful and no interaction between the 5-FU and hydrogel ingredients was detected [34].

### 2.4. Swelling Studies

The release of the drug from the 5-FULH occurs once the polymer network is dissolved, followed by drug diffusion from the surface of the structure which is associated with the swelling behavior of the 5-FULH [35]. The advantage of hydrogel is that it can swell in the surrounding medium due to its chemical structure, which allows affinity with the water molecules [36]. Swelling studies of 5-FULH were conducted using various pH mediums of 1.2, 6.8, and 7.4. Equilibrium and dynamic swelling proportions of different 5-FULH are depicted in Table 2.

### 2.5. Effect of AM

**Figure 2:**
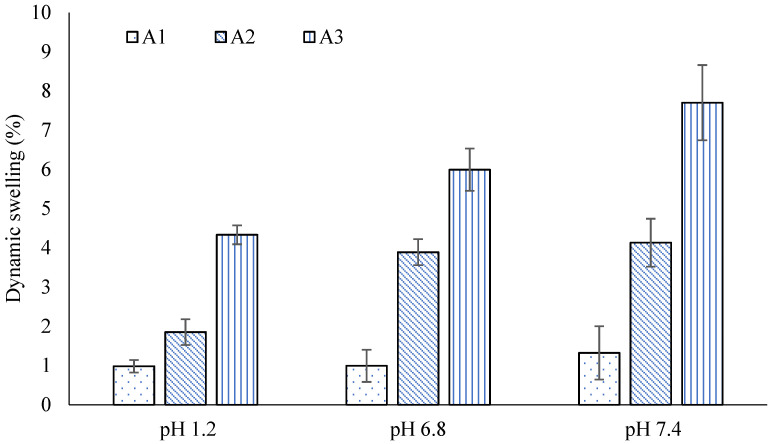
Effect of AM on dynamic swelling of 5-FULH.

**Figure 3:**
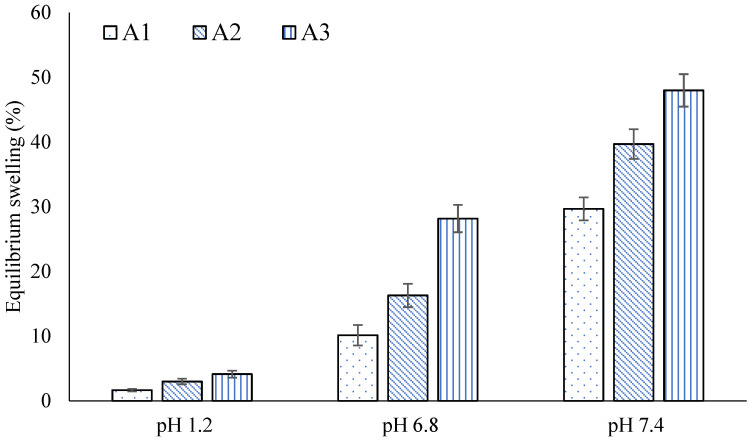
Effect of AM on equilibrium swelling of 5-FULH.

Figure 2 and Figure 3 describe the swelling attitude of 5-FULH with various concentrations of AM. The swelling values of these gels were measured at 37 °C and medium pH of 1.2, 6.8, and 7.4. The swelling increased with an increase in AM concentration and a raise in medium pH. This swelling behavior of the polymer was due to its hydrophilic nature. The physical nature and hydrogen bond formation restricts the release of the drug.

### 2.6. Effect of AA

**Figure 4:**
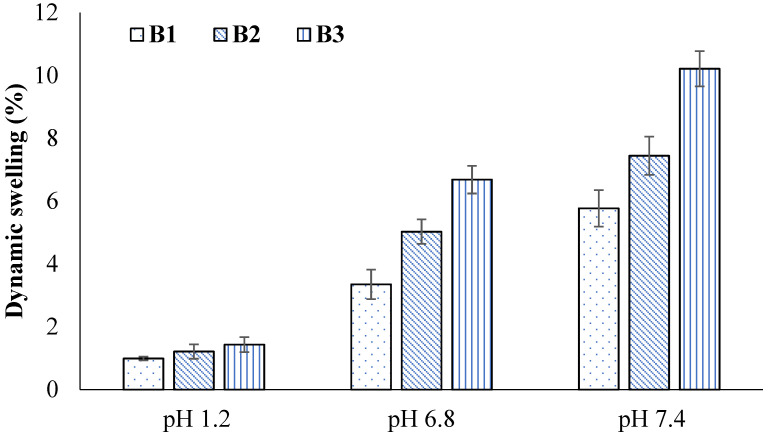
Effect of AA on dynamic swelling of 5-FULH.

**Figure 5:**
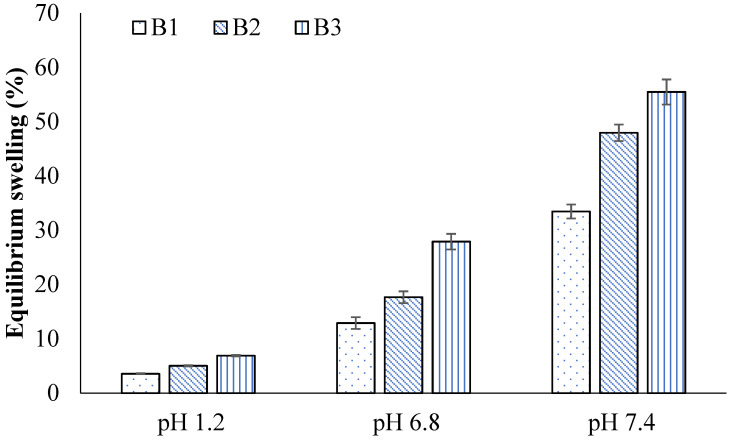
Effect of AA on equilibrium swelling of 5-FULH.

Figure 4 and Figure 5 show the swelling attitude of 5-FULH with various concentrations of AA. The swelling values of these gels were measured at 37 °C and medium pH of 1.2, 6.8, and 7.4. The swelling increased with an increase in AA concentration and a raise in medium pH. This swelling behavior of the polymer was again due to its hydrophilic nature. The physical nature and hydrogen bond formation again restricts the release of the drug.

### 2.7. Effect of GA

**Figure 6:**
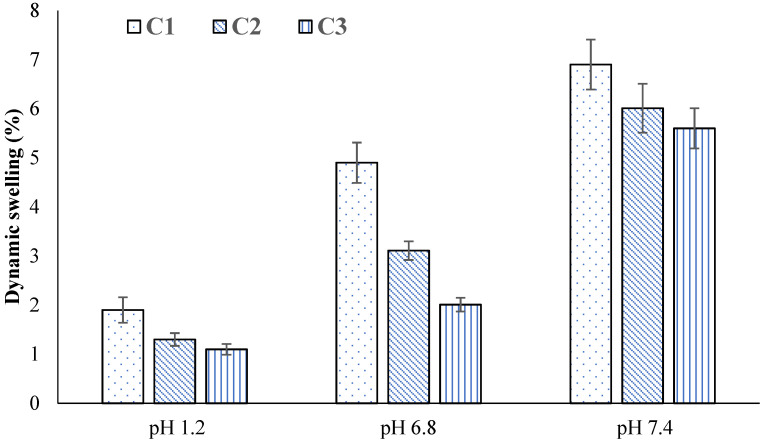
Effect of GA over dynamic swelling of 5-FULH.

**Figure 7:**
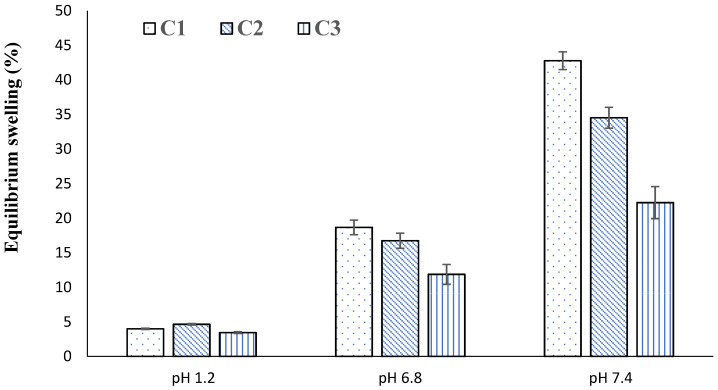
Effect of GA over the equilibrium swelling of 5-FULH.

Introduction of a crosslinking agent such as GA affects the swelling behavior of 5-FULH. Figure 6 and Figure 7 show GA at different concentrations and the effect over the swelling of hydrogel at 37 °C in medium pH of 1.2, 6.8, and 7.4. Results show that when the concentration of GA increases, the swelling ratio decreases. This might be due to increased cross linking of GA. Generally, mobility of the polymer chain is affected by the crosslinking, ensuring the low solubility of the polysaccharide [37]. GA promotes the degree of crosslinking which in turn results in folding of the polymeric chains and the subsequent attainment of reticulation point, thus affecting the aqueous absorption capacity [38].

### 2.8. Porosity Measurement

The porosity values of 5-FULH range from 34% to 62% with different weight ratios of AM, AA, and GA. It was found that the higher the concentration of polymer and monomer, the higher the porosity values. While enhancing the crosslinker concentration, the porosity values decrease. Table 3 (B3) shows a higher % of gel fraction (94 ± 0.2%) with a higher % of porosity (62 ± 0.06%); a similar type of relationship was found in previous studies. The controlled release of the drug, around 65% at 25 h, could be attributed to the reduction of water entry and subsequent diffusion of the drug from the hydrogel network [39].

### 2.9. Gel Fraction

Table 3 shows the results of gel and sol fraction. In relation to the value of gel fraction, it was observed that the gel fraction depended on the AM and AA. The gelling strength of the prepared 5-FULH may increase with the higher content of AM, AA, and GA. As the concentration of the AA is increased, the polymerization reaction is also enhanced due to the accessibility of more binding sites. The higher gel fraction is attributed to the increased bulk density of the hydrogel structure. A firm and robust hydrogel network is established due to the higher concentration of polymer, resulting in a greater degree of crosslinking which in turn leads to a lower porous structure of hydrogels [40]. However, sol fraction is decreased since the concentration of AA and GA is increased due to the inverse relationship with gel fraction [41].

### 2.10. Drug Loading

Drug loading was determined by using swelling, extraction, and weight values. The amount of 5-FU was presented as g/g of dry gel and the data were presented in Table 4.

### 2.11. Cumulative Drug Release Measurement

From the polymeric 5-FULH, it can be seen that the release pattern of a formulation depends on the swelling behavior of these hydrogels. This section discusses some of the factors which influence the release of 5-FU from the prepared hydrogels.

### 2.12. Effect of AM

**Figure 8:**
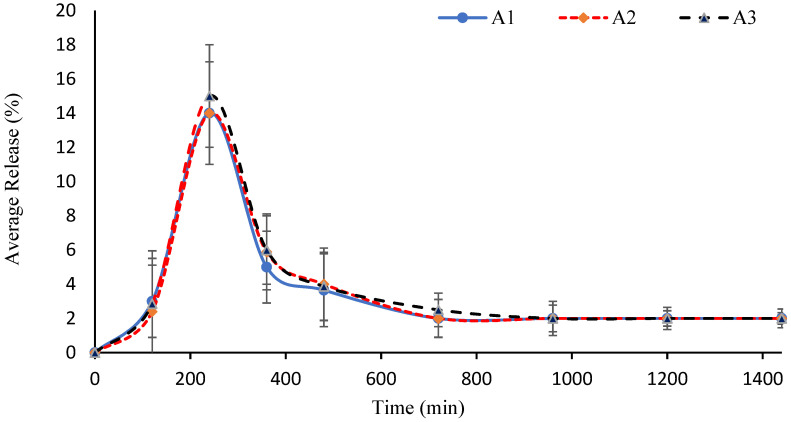
At pH 1.2, effect of AM on 5-FU release.

**Figure 9:**
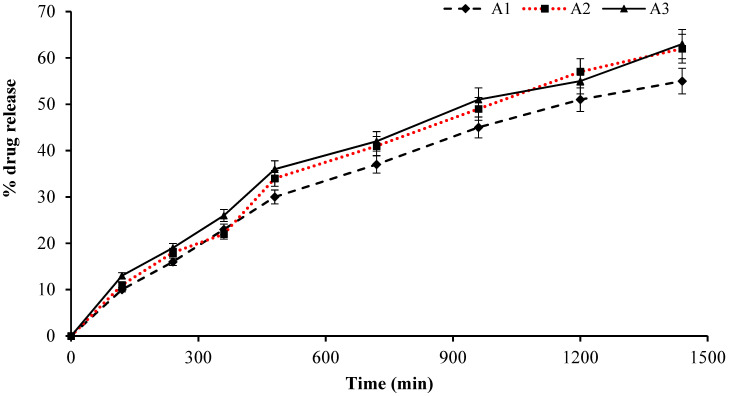
At pH 7.4, effect of AM on 5 FU release profile.

These 5-FULH were designed by varying AM concentrations at 37 °C and pH of 1.2 and 7.4. The release of 5-FU is very low in an acidic medium, increasing to 67% in a basic medium. This might be due to greater swelling in a basic medium and less swelling in an acidic medium. Mundargi et al. reported that the release of 5-FU increases when AM content increases in the matrix [42]. Figure 8 shows that drug release was varied based on the pH of the medium i.e., around 20% drug release was found at 1 h in an acidic media (pH 1.2), whereas >65% of drug release (pH 7.4) was observed at around 25 h from or with? the 5-FULH containing a higher amount of AM due to increased polymer chain flexibility, as shown in Figure 9. This release behavior of the drug was in agreement with the previous study where single polymeric chains in pure AM allowed higher drug transportation compared to the co-polymeric chain [43].

### 2.13. Effect of AA

**Figure 10:**
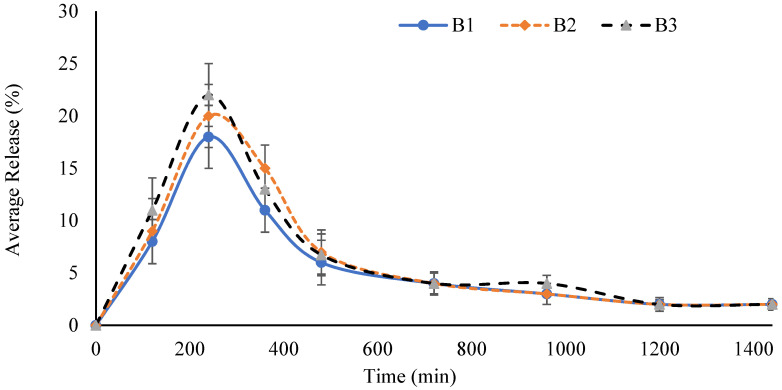
Effect of AA on the release profile of 5-FU at pH 1.2.

**Figure 11:**
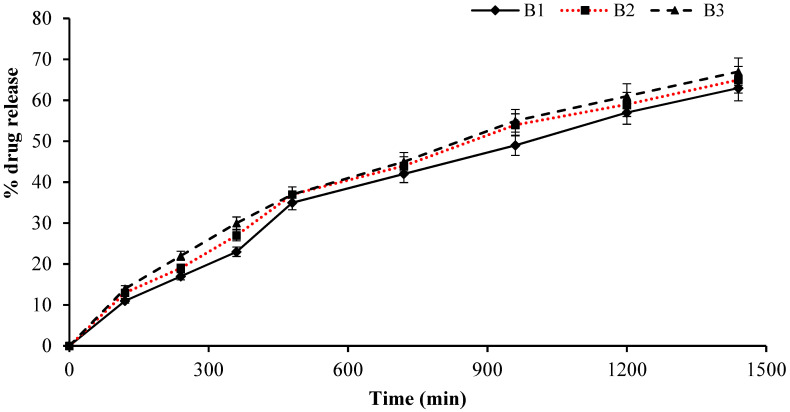
Effect of AA on the release profile of 5-FU at pH 7.4.

Figure 10 and Figure 11 present the release profile of 5-FU under the influence of AA. The higher the concentration of AA, the higher the release of 5-FU. Enhancing the quantity of polymer AA increases the release rate of 5-FU due to the development of channels and pores in the hydrogel matrix resulting from relaxation of the polymer network. This result is in agreement with previous studies where drug release was enhanced with the higher pH and the amount of AA [44].

### 2.14. Effect of GA

**Figure 12:**
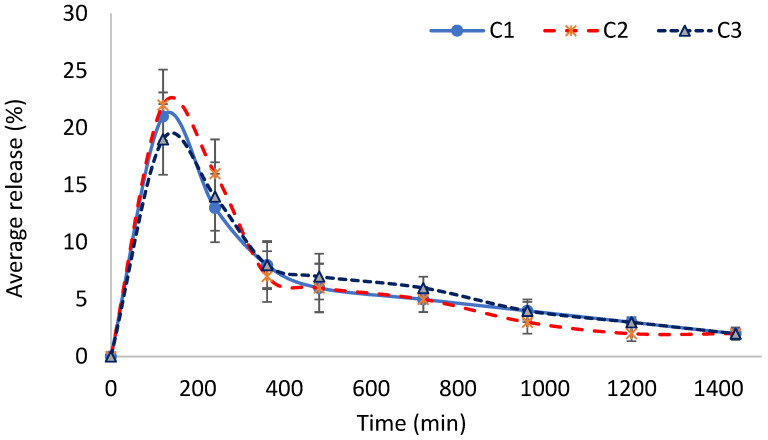
Effect of GA on the release profile of 5-FU at pH 1.2.

**Figure 13:**
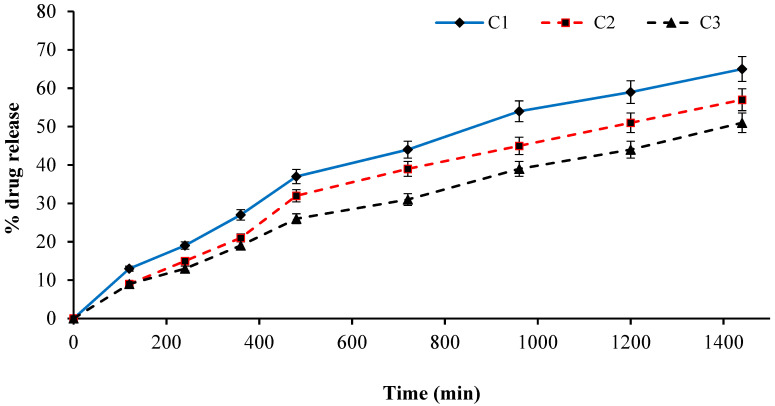
Effect of GA over 5-FUrelease profile at pH 7.4.

Figure 12 and Figure 13 describe the release of 5-FU under different concentrations of GA. The release of 5-FU in an acidic medium was almost the same irrespective of different concentrations of GA. The release of 5-FU was lower with a higher concentration of GA when it comes to basic medium pH 7.4. This could be attributed to the higher H-bonding and lower swelling of 5-FULH with a high concentration of crosslinking agent. It has been reported that the gelation ratio is faster in GA crosslinked hydrogel, therefore higher GA concentration increases the drug loading capacity [45]. As seen in Figure 13, the 5-FU release percentages from the 5-FULH at pH 7.4 were more than 50% of the pH 1.2 at 18% in every formulation. The present findings are in line with the previous data which investigated 5-FU loaded GA blended pH-responsive hydrogel and found 5-FU release of around 64.0% to 85.99% at pH 7.4 but only 13.33% to 19.64% at pH 1.2 [46].

The protonation of sulfonate ions occurs at acidic pH 1.2, which results in the generation of strong hydrogen bonds and physical interaction between the functional groups of the hydrogel, causing a subsequent reduction in swelling [47]. However, at pH 7.4, deprotonation of sulfonate ions occurs, owing to the enhanced electronic density on the polymeric structure and less physical interaction among the –SO^−3^ moieties, thereby generating a greater degree of swelling of AA 5-FULH [48]. All of these factors cause reduction in intermolecular hydrogen bonding, resulting in higher swelling [49]. Likewise, the COOH groups of AA are protonated at pH 1.2 which leads to a fall in swelling. However, at higher pH 7.4, carboxylic acid (CA) functionalities are deprotonated, resulting in higher swelling of the 5-FULH [50]. Also, the osmotic pressure of the ions is increased due to the increase in protonation of the COOH groups [51]. The carboxylic acid (CA) moieties were converted to the salt form which led to maximum swelling, as the pH of the medium was elevated from a lower to a higher number [52].

### 2.15. Release Kinetics

Several kinetic models were employed to help understand the release mechanism of the 5-FU from the hydrogels, and the regression coefficients (*r*) were selected for the evaluation of the most suitable drug release [53]. The (*r*) values of most samples were greater for zero-order kinetics compared to the first-order kinetics, which could be ascribed to the variation in the amounts of AA and GA. From the results of the Higuchi model, it was obvious that the release of the drug follows the diffusion-controlled release mechanism. Among the models, the (*r*) values of 5-FULH for zero order (0.9632–0.9954) was comprehensively higher than the first order (0.9378–0.9932). The results indicated that the release pattern of the drug corresponded to the zero-order kinetics.

## 3. Conclusions

The 5-FULH were processed for oral delivery using AM, AA and GA crosslinked. These prepared 5-FULH were physio-chemically characterized. ATR-FTIR shows no such interaction between the excipients and the model drug (i.e., 5-FU). Swelling studies show that minimum swelling was achieved at acidic pH and maximum at alkaline Ph, which depends on the monomer, polymer, and crosslinking agent. By increasing AM and AA concentrations, swelling increased, whereas by increasing GA, it decreased. In vitro release studies show that the most drug was released on alkaline pH, whereas the least drug was released on acidic pH. The release of the drug also increases with increasing AM and AA content but decreases with an increase in cross linker. The drug release kinetics models followed a non-Fickian order of release. These findings concluded that AM and AA based 5-FULH would be appropriate for controlled drug delivery with pH reactive characteristics.

## 4. Materials and Methods

### 4.1. Materials

5-Fluorouracil (5-FU) was obtained from Biolabs Pharma Pvt. Ltd., (Biolabs Pharma, Islamabad, Pakistan). Acrylamide (AM), acrylic acid (AA), glutaraldehyde (GA), disodium hydrogen phosphate, sodium hydroxide, and monobasic potassium phosphate were purchased from Sigma Aldrich (Sigma Aldrich, Darmstadt, Germany). Hydrochloric acid and acetic acid were supplied by Icon Chemicals (Icon Chemicals, Ludhiana, Punjab, India).

### 4.2. Pre-Formulation Studies and Standard Curve

The stock solution was prepared using 10 mg of 5-FU dissolved in 100 mL of phosphate buffer pH 7.4. A series of dilutions were made in descending order using phosphate buffer pH 7.4. Samples from such mixtures were taken and spectrophotometrically analyzed at 256 nm (Shimadzu 601, Japan). The results were taken in triplicate and plotted.

(1) Y=MC+B

where:

*Y* = the absorbency of the solution, *M* = the angle of standard curve of identified concentration, *C* = concentration that must be calculated and *B* = the curve’s cut off.

### 4.3. Solubility Study of Drug

The solubility study of 5-FU was conducted using different solvents of varying pH at 37 °C.

### 4.4. Preparation of 5-FULH

The 5-FULH were produced by a simple crosslinking method, with slight modifications as presented in Table 5. In the first step a specific amount of distilled water was put into a beaker and placed on a magnetic stirrer and heated. Thereafter the AM was poured into the beaker and heated up till a clear solution was achieved. Similarly, by applying slight modifications in temperature as well as in revolution time, an AA solution was obtained. These solutions were then thoroughly mixed with continuous stirring on a magnetic stirrer until a clear homogeneous liquid was produced. Finally, by adding distilled water, the volume of the solution was made up to a hundred milliliters. Consequently, in a drop wise method and with regular stirring, cross linker was added to the mixture, to produce a uniform clear solution. This solution was then poured into the deoxygenized test tubes and left to congeal in them for four days. The 5-FULH, once they were formed, were then removed from the tubes. With the help of sharpened blades, these gels were cleaved as well as properly scrubbed with Methanol. The discs were then put into petri dishes and desiccated in an oven at 40 °C for seventy-two hours [54,55].

### 4.5. Hydrogel’s Characterizations

#### 4.5.1. ATR-FTIR

Physical interactions between drug and excipients were investigated using ATR-FTIR (Perkin Elmer, Waltham, MA, USA). The samples were placed on ATR-FTIR and scanned from 400–4000 cm^−1^ [56].

#### 4.5.2. Dynamic Swelling Study

Dried slices of these preparations were properly weighed and immersed into buffers of various pH (i.e., pH 7.4, pH 6.8, and pH 1.2). By removing discs from the medium, readings were noted over regular time periods [57,58]. The surface water was removed from the 5-FULH using tissue paper. The discs were dipped again in the pH 6.8 and 7.4 buffer, after having been weighed at definite intervals [59]. The following formula was used to obtain the dynamic *S*:

(2) $$S=\frac{Ws}{Wd}$$

where:

*Ws* = weight of swollen gel at specific time, *Wd* = weight of dry hydrogel, and *S* = swelling ratio.

#### 4.5.3. Equilibrium Swelling Study

*ES* studies were also performed on the 5-FULH, which were prepared for frequent swelling. The % *ES* was determined using the following equation [60,61].

(3) $$ES\;\left(\%\right)=\frac{Ws-Wd}{Wd}\times 100$$

where:

*ES* = equilibrium swelling, *Ws* = weight of swollen gel at specific time, and *Wd* = weight of dry hydrogel.

#### 4.5.4. Diffusion Coefficient (DC)

DC is the quantity of solvent that was absorbed/diffused across the unit area in unit time through a concentration gradient [62,63]. The diffusion coefficient was calculated by Formula (4):

(4) $$D=\pi {\left(\frac{h\cdot \theta }{4\cdot Qeq}\right)}^{2}$$

where:

*D* = DC of the 5-FULH, *Qeq* = the hydrogel’s swelling at equilibrium, *θ* = the angle of straight portion of swelling curves and *h* = early sample thickness.

#### 4.5.5. Sol Gel Fraction Analysis

Sol-gel analysis technique was performed for predisposing reactant quantity used during the preparation of the 5-FULH. The prepared 5-FULH were dried without washing and placed in deionized water at ambient temperature until constant weight was attained. After that, extracted hydrogel was taken out and dried in an oven at 60 °C [64,65].

(5) $$\mathrm{Sol}\;\mathrm{fraction}\;(\mathrm{SF})\;(\%)=\frac{M_{2}-M_{1}}{M_{1}}\times 100$$

where:

*M*_2_ = Final/extracted gel wt, *M*_1_ = Initial wt of dry gel and

(6) Gel fraction=100−SF

#### 4.5.6. Porosity Measurement

Porosity is an important consideration mainly affecting the swelling attributes of the 5-FULH. The % porosity was calculated by solvent replacement technique. Hydrogel discs were dried and soaked in ethanol (100%) overnight. Extra ethanol was removed using blotting paper and then the 5-FULH were weighed [66]. The porosity was calculated and attained by using the following equation:

(7) $$\mathrm{Porosity}\;(\%)=\frac{M_{t}-M_{o}}{\rho v}\times 100$$

where:

*M_t_* = weight before immersion, *M_o_* = weight after immersion, *ρ* = density of absolute ethanol and *v* = volume of hydrogel.

#### 4.5.7. Drug Loading

Samples which showed maximum swelling were used for drug loading and release studies. The drug loading into the discs of hydrogel was achieved by soaking them for one week in a solution of the drug. A 1% *w*/*v* 5-FU in pH 7.4 solution was used for drug loading. After achieving the equilibrium value, the swelled 5-FULH were removed from the drug solution, blotted with filter paper, first dried at room temperature, and then placed in an oven at 40–45 °C for one week to remove the absorbed solvent. To determine the percentage of drug-loading, weighed drug loaded samples were extracted repeatedly using a phosphate buffer solution of pH 7.4 up to exhaustion, and the concentration of the drugs in pooled extract was determined spectrophotometrically at λ max 256 nm. The quantity of drug loaded into the 5-FULH was also determined by the swelling method [67].

#### 4.5.8. In Vitro Release Study

To assess the amount of released drug from formulated 5-FULH, in vitro release studies were performed, using dissolution apparatus (pharma test; Pt-Dt 7). These hydrogel discs were positioned in a dissolution medium of 500 mL at 37 °C temp in a pH of 1.2 and 7.4 and shaken at a hundred revolutions per minute. Samples were selected at specific time periods and replaced. These samples were then evaluated at 256 nm using a UV-spectrophotometer (Shimadzu) [68].

#### 4.5.9. Release Kinetics

To investigate the discharge of medicament from the gels and its mechanism, various kinetic models were applied [58], which are given below.
First order kineticsln (1 − F) = K1tZero order kineticsM_o_ − M_t_ = K_o_tHiguchi modelQ = K_2_t^1/2^Korsmyer Peppas ModelMt/Mœ = Kt^n^

### 4.6. Statistical Analysis

Findings from all of the experiments were made in triplicate and presented as the means ± S.D. These samples were analyzed statistically with the help of one-way ANOVA and *t*-test. At *p* < 0.05, differences were considered as significant.

## Figures and Tables

**Table 1:** Solubility study of 5-FU at 37 °C.

S.No	Solvent	Remarks
1	Distilled Water	Soluble
2	Phosphate buffer pH 7.4	Soluble
3	Acidic media pH 1.2	Sparingly soluble

**Table 2:** % Dynamic and equilibrium swelling study of 5-FULH.

F. Code	pH 1.2		pH 6.8		pH 7.4	
	Dynamic Swelling	Equilibrium Swelling	Dynamic Swelling	Equilibrium Swelling	Dynamic Swelling	Equilibrium Swelling
A1	0.98 ± 0.04	1.65 ± 0.01	1.85 ± 0.01	10.12 ± 0.02	4.33 ± 0.04	29.67 ± 0.05
A2	0.99 ± 0.03	2.98 ± 0.03	3.89 ± 0.03	16.27 ± 0.03	5.99 ± 0.03	39.68 ± 0.03
A3	1.32 ± 0.01	4.11 ± 0.05	4.13 ± 0.05	28.15 ± 0.01	7.71 ± 0.03	47.98 ± 0.02
B1	0.99 ± 0.02	3.55 ± 0.02	3.35 ± 0.02	12.89 ± 0.04	5.77 ± 0.02	33.46 ± 0.07
B2	1.21 ± 0.04	5.02 ± 0.01	5.02 ± 0.01	17.65 ± 0.05	7.44 ± 0.03	47.96 ± 0.05
B3	1.43 ± 0.02	6.88 ± 0.03	6.68 ± 0.03	27.89 ± 0.03	10.21 ± 0.01	55.48 ± 0.04
C1	1.90 ± 0.03	3.99 ± 0.01	4.99 ± 0.01	18.67 ± 0.02	6.88 ± 0.02	42.78 ± 0.06
C2	1.33 ± 0.01	4.64 ± 0.02	3.14 ± 0.02	16.74 ± 0.01	6.10 ± 0.03	34.53 ± 0.05
C3	1.10 ± 0.01	3.45 ± 0.04	2.05 ± 0.04	11.87 ± 0.04	5.66 ± 0.02	22.25 ± 0.03

**Table 3:** Diffusion coefficient, porosity %, gel fraction %, and sol fraction % of formulated 5-FULH.

F. Code	DC (cm^2^/s)	Porosity %	Gel Fraction %	Sol Fraction %
A1	0.19 ± 0.02	34 ± 0.03	74 ± 0.4	24 ± 0.1
A2	0.09 ± 0.03	42 ± 0.04	86 ± 0.3	22 ± 0.2
A3	0.05 ± 0.01	55 ± 0.02	90 ± 0.4	15 ± 0.1
B1	0.08 ± 0.01	41 ± 0.03	85 ± 0.5	17 ± 0.3
B2	0.07 ± 0.03	43 ± 0.04	91 ± 0.3	11 ± 0.2
B3	0.03 ± 0.02	62 ± 0.06	94 ± 0.2	09 ± 0.3
C1	0.15 ± 0.01	39 ± 0.05	83 ± 0.4	16 ± 0.4
C2	0.09 ± 0.03	39 ± 0.04	89 ± 0.4	11 ± 0.4
C3	0.07 ± 0.02	47 ± 0.03	93 ± 0.3	09 ± 0.3

**Table 4:** Amount of 5-Flurouracil loaded in different formulations of acrylamide/acrylic acid hydrogels.

F. Code	Amount of 5-FU Loaded (g/g of Dry Gel)		
	By Swelling	By Extraction	By Weight
A1	0.0394	0.0373	0.0347
A2	0.0402	0.0392	0.0369
A3	0.0416	0.0423	0.0391
B1	0.0372	0.0393	0.0357
B2	0.0349	0.0385	0.0341
B3	0.0331	0.0377	0.0328

**Table 5:** Composition of formulated 5-FULH.

F. Code	AM	AA	GA	TEMED	APS	D/Water
A1	4.08 g	0.48 g	23 µL	235 µL	0.20 g	13 mL
A2	5.88 g	0.48 g	23 µL	235 µL	0.20 g	13 mL
A3	7.5 g	0.48 g	23 µL	235 µL	0.20 g	13 mL
B1	4.08 g	0.96 g	23 µL	235 µL	0.20 g	13 mL
B2	4.08 g	1.5 g	23 µL	235 µL	0.20 g	13 mL
B3	4.08 g	2 g	23 µL	235 µL	0.20 g	13 mL
C1	4.08 g	0.48 g	19 µL	235 µL	0.20 g	13 mL
C2	4.08 g	0.48 g	13 µL	235 µL	0.20 g	13 mL
C3	4.08 g	0.48 g	7 µL	235 µL	0.20 g	13 mL

## Data Availability

Data will be available upon request.
